# Supplementary material for: Optimized single-step optical clearing solution for 3D volume imaging of biological structures
Source: Commun Biol. 2022 May 9;5:431. doi: 10.1038/s42003-022-03388-8 (PMC9085829; doi:10.1038/s42003-022-03388-8)
Supplement: Supplementary file 2 — Supplementary Information [file 42003_2022_3388_MOESM2_ESM.pdf]

Supplementary Information for

Optimized single-step optical clearing solution for 3D volume imaging of biological structures

Kitae Kim[1], Myeongsu Na[1], Kyoungjoon Oh[1], Eunji Cho[1], Seung Seok Han[2] and Sunghoe Chang[1,3]*

[1]Department of Physiology and Biomedical Sciences, Seoul National University College of Medicine , Seoul 03080, South Korea

[2]Department of Internal Medicine, Seoul National University Hospital, Seoul 03080, South Korea.

[3]Neuroscience Research Institute, Seoul National University College of Medicine, Seoul 03080, South Korea

* corresponding author e-mail address: sunghoe@snu.ac.kr

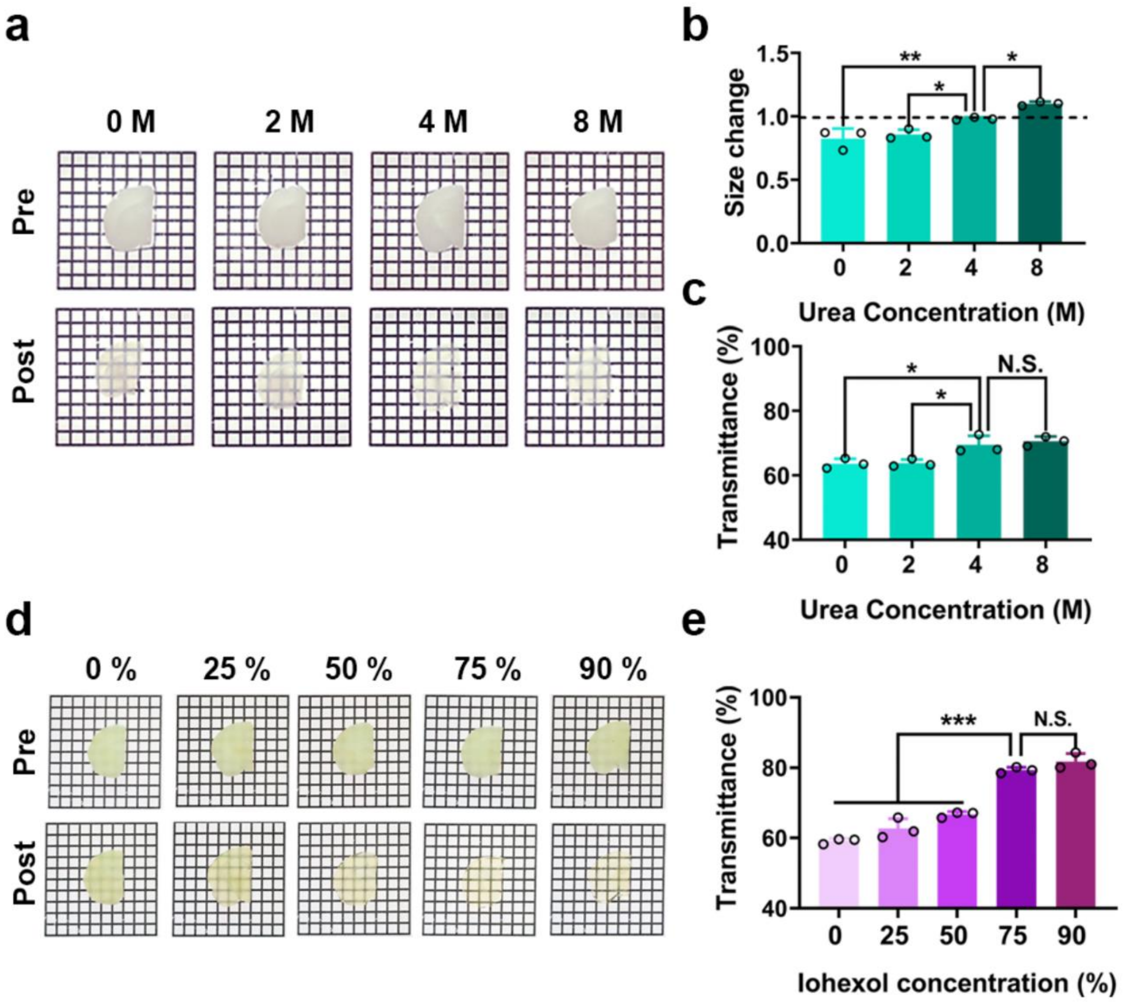

Supplementary Figure 1. Optimization of urea and iohexol concentration for OptiMuS.

(a) Bright-field images of pre- and post-cleared 1-mm thick rat brain slices at various urea concentrations. Grid size = 1.5 x 1.5 mm. (b) Quantification of size change over urea concentrations. (c) Quantification graph of transmittance over urea concentration. (d) Bright-field images of pre- and post-cleared 1-mm thick rat brain slices at various iohexol concentrations. (e) Quantification graph of transmittance over iohexol concentration. Grid size = 1.5 x 1.5 mm, (n = 3). The data are shown as the mean ± SD. *** $p<0.001$, ** $p<0.01$, * $p<0.05$.

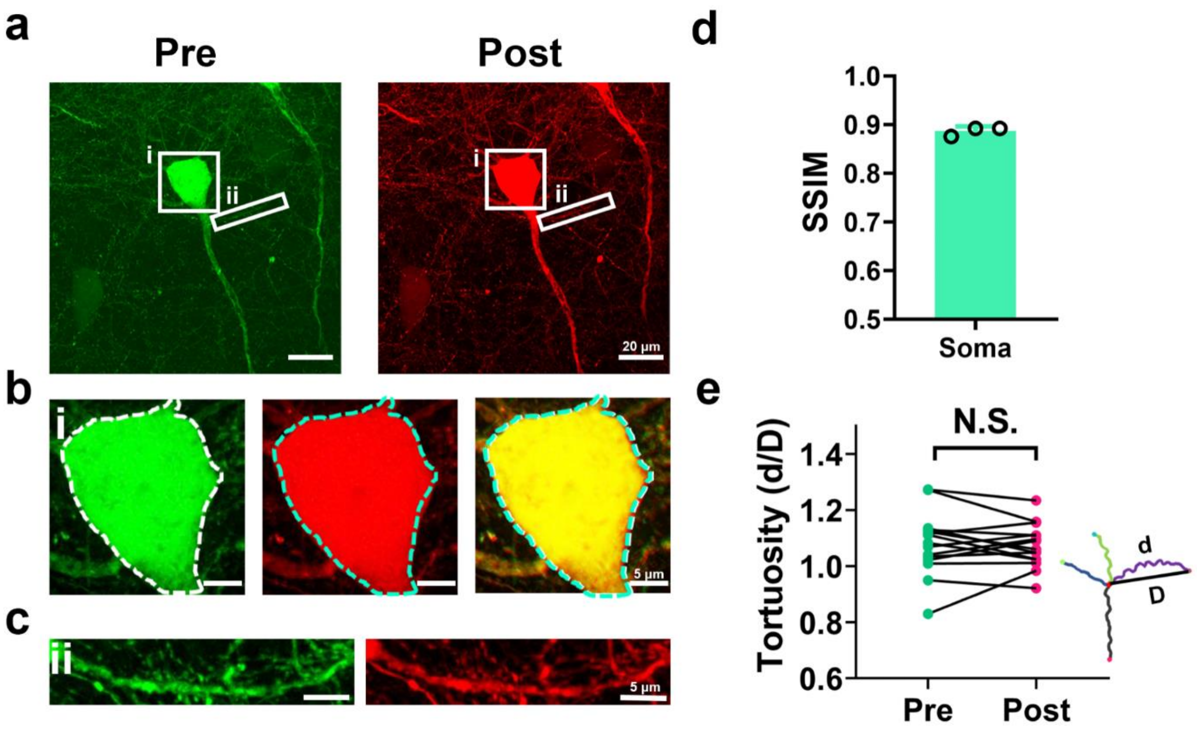

Supplementary Figure 2. OptiMuS preserves neuronal ultrastructures.

(a) Maximum z-projection fluorescence image of 50-μm thick *Thy1*-EYFP brain slice before (pre-) and after (post-) cleared by OptiMuS. (b) Magnified images of soma in (a)-i; pre (left), post (middle), merge (right). (c) Magnified images of dendrite segment in (a)-ii; pre (left), post (right). (d) Structural Similarity Index Measure (SSIM) between pre- and post- somas shown in (b). (n = 3). (e) Comparison of tortuosity between pre- and post-cleared dendrite segments. The minimum distance (D) and actual length (d) between the two ends of each individual dendrite. Morphological change was expressed by calculating the torsional index ratio (d/D) of each pre and post. The data were shown as the mean ± SD. *** $p<0.001$, ** $p<0.01$, * $p<0.05$.

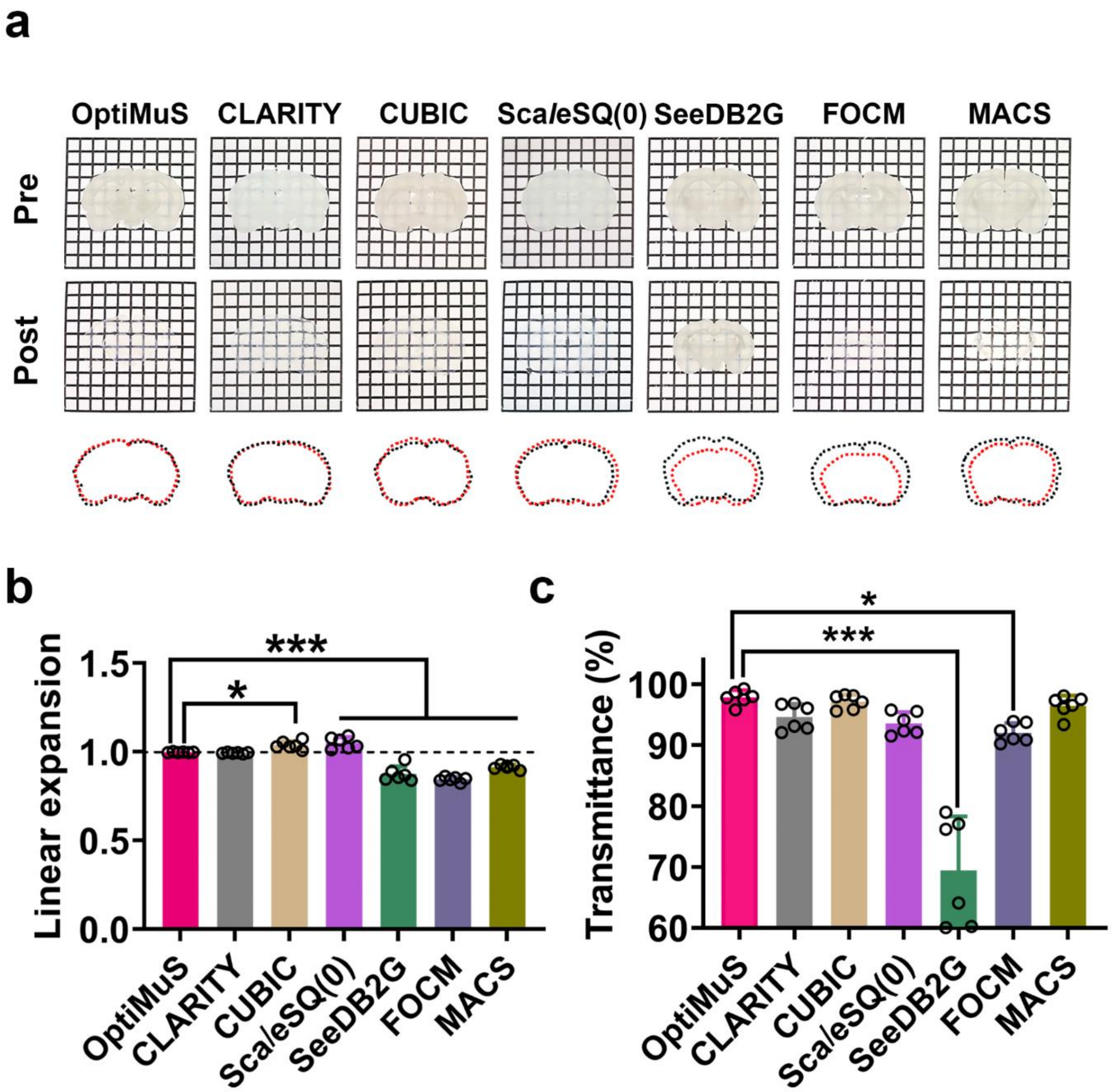

Supplementary Figure 3. OptiMuS also provides the best clearing performance in a thin brain section.

(a) Bright-field images of 200-µm rat brain samples before (pre-) and after (post-) cleared by OptiMuS, CLARITY, CUBIC, Sca*l*eSQ, SeeDB2G, FOCM and MACS. Grid size = 1.5 x 1.5 mm. (b) Quantitative comparison of the linear expansion after each clearing method. (clearing time: 2 min; n = 6). (c) Quantification of transmittance of each clearing method (n = 6). The data were shown as the mean ± SD. *** p<0.001, ** p<0.01, * p<0.05.

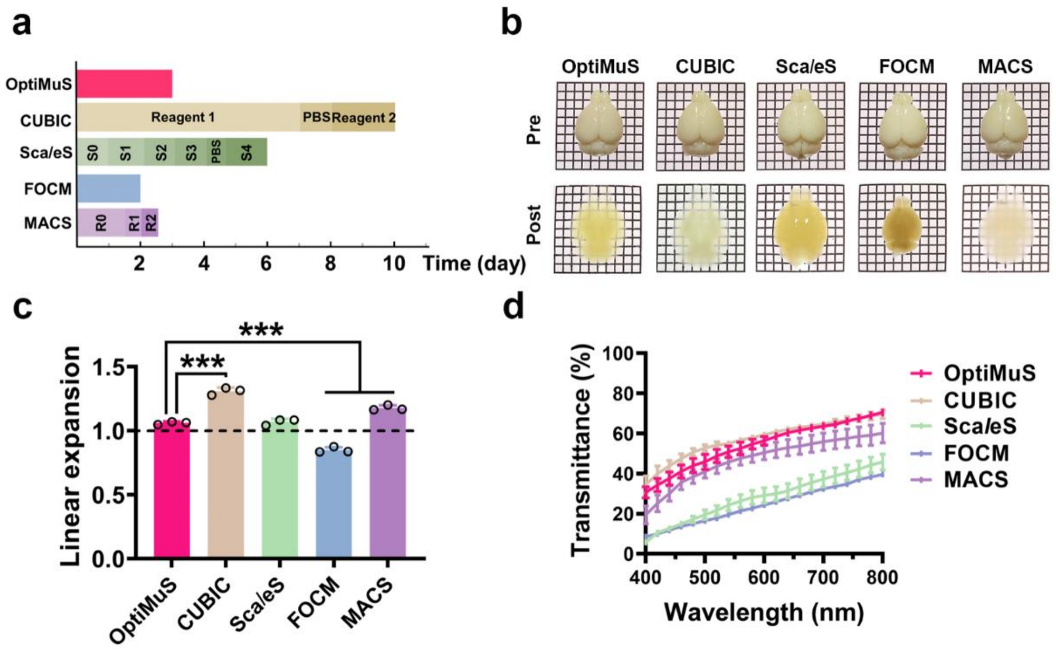

Supplementary Figure 4. OptiMuS also provides optimal clearing performance in the whole mouse brain

(a) The time line of OptiMuS, CUBIC, Sca*l*eS, FOCM and MACS for clearing of the whole mouse brain. (b) Bright-field images of pre- and post-cleared by each method in (a). (c) Quantification of the linear expansion after each clearing method (n = 3). (d) Transmittance scan curves at 400 ~ 800 nm after clearing of the whole mouse brain by each method (n = 3). The data were shown as the mean ± SD. *** $p<0.001$, ** $p<0.01$, * $p<0.05$.

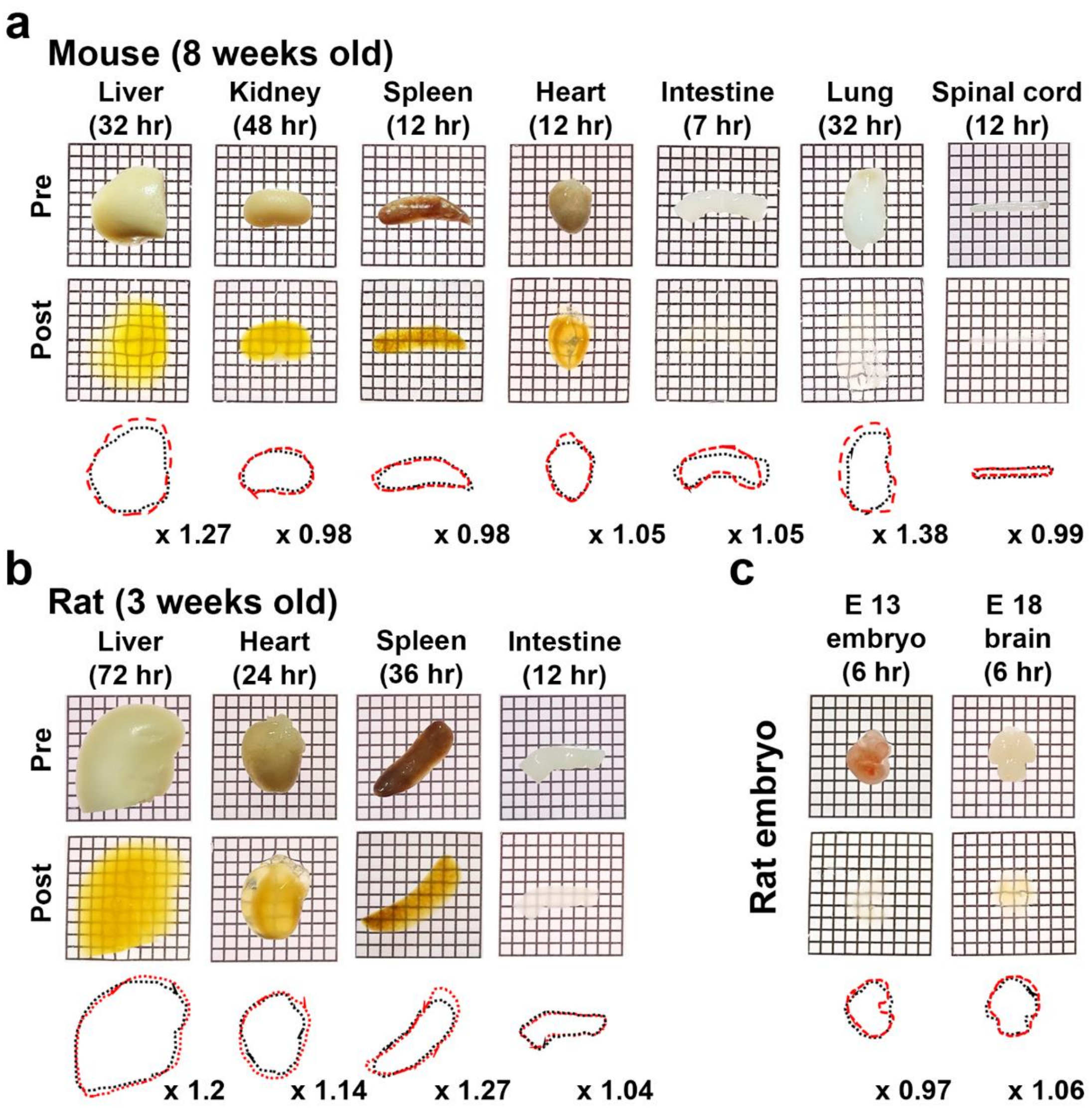

Supplementary Figure 5. OptiMuS effectively rendered high transparency of various mouse and rat organs including E-13 embryo and E-18 embryonic brain.

(a) Bright-field images of 8-week old various mouse organs pre- and post-cleared by OptiMuS. (b) Bright-field images of 3-week old various rat organs pre- and post-cleared by OptiMuS. (c) Bright-field images of E-13 rat embryo and E-18 rat embryonic brain pre- and post-cleared by OptiMuS. The number in parentheses indicates the clear time for each organ. Overlapped images of the outlined pre- and post-cleared organs and the ratio of areas between pre-and post-cleared images. (black: pre-, red: post-cleared). Grid size = 1.5 x 1.5 mm.

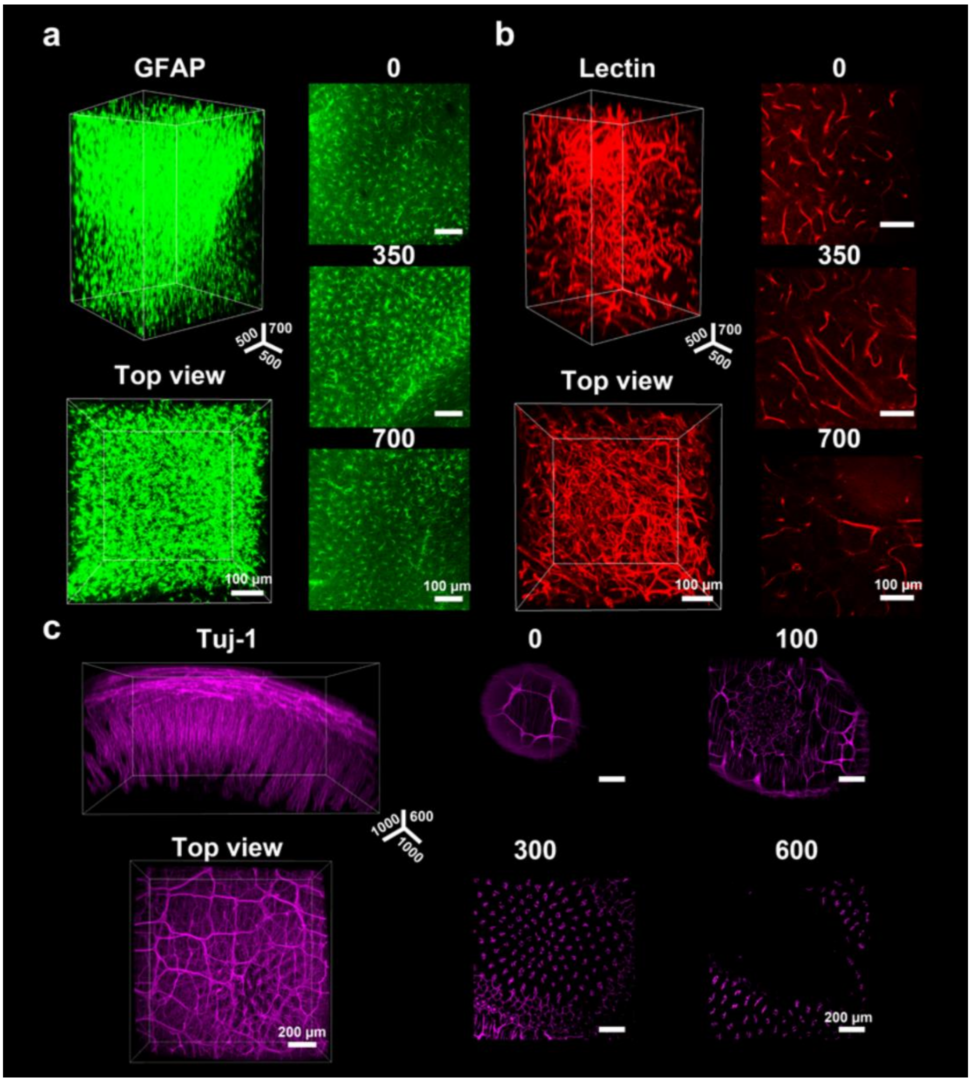

Supplementary Figure 6. OptiMuS enables 3D volumetric imaging of neural and vascular structures in the samples that were pre-stained with various antibodies and lectin.

3D reconstruction images of mouse brain samples that were pre-stained with anti-GFAP (glial fibrillary acidic protein) antibody (a) or fluorophore-conjugated Lycopersicon esculentum (tomato) lectin, a blood vessel marker (b) to visualize neural or vascular structures. Scale bar = 100 µm. (a) staining of mouse brain sample (700-µm thick). (b) staining of mouse brain sample (700-µm thick). (c) 3D reconstruction images of mouse intestine sample that was stained with anti-Tuj-1 (neuron-specific class III beta-tubulin) antibody. Scale bar = 200 µm. Each optical section at various depths (right). The 3D axis is expressed in µm.

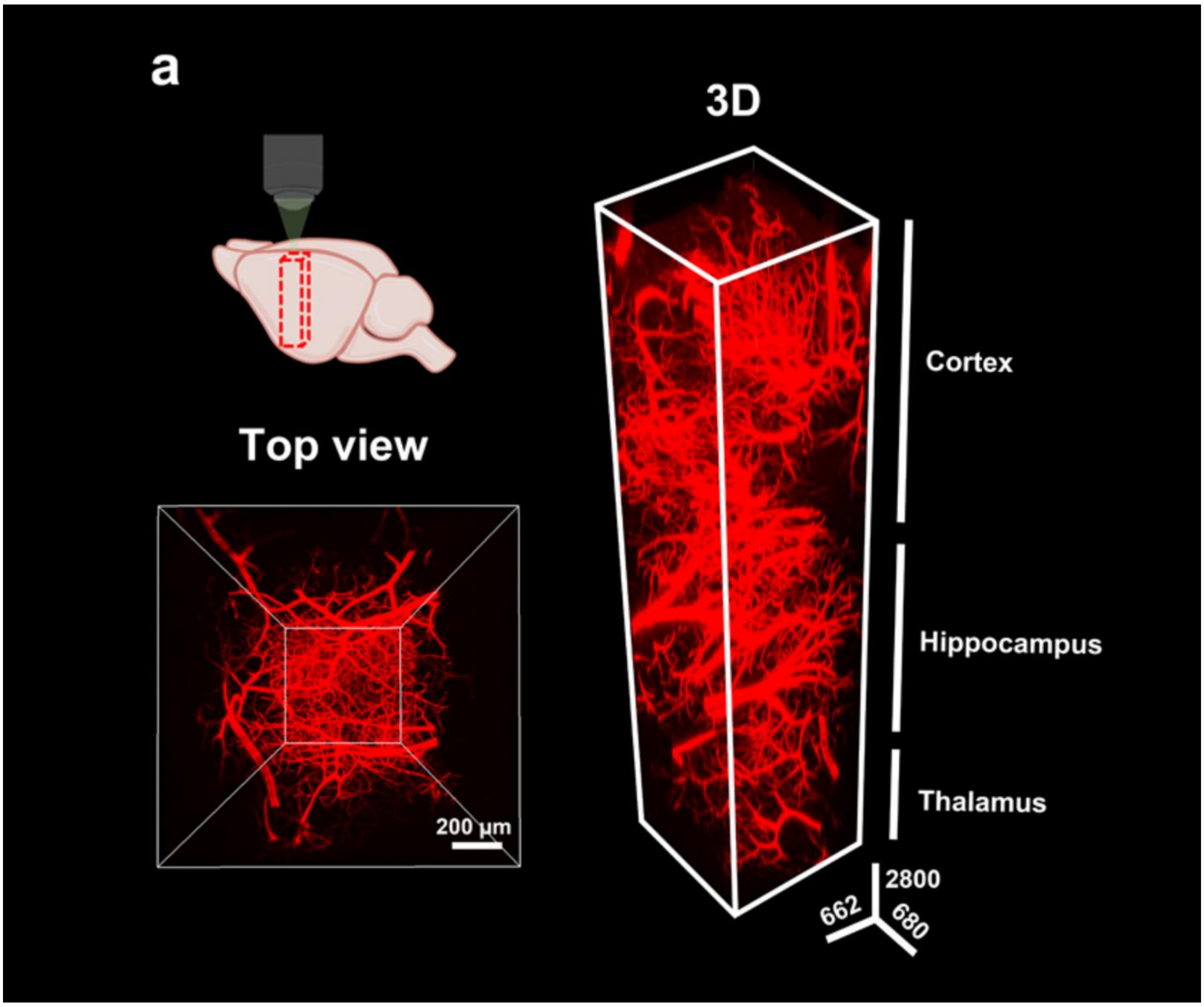

Supplementary Figure 7. OptiMuS visualizes DiI-labeled vascular structures in the brain after the transcardial perfusion.

Fluorescence image of 2,800-μm thick DiI-labeled brain sample. DiI was transcardially perfused before tissue isolation and sectioning, and images were taken with an upright confocal microscope with a 10x lens (NA = 0.5, WD = 5.5 mm). The top view image and 3D reconstruction image with the corresponding brain regions. Scale bar = 200 μm. The 3D axis is expressed in μm.

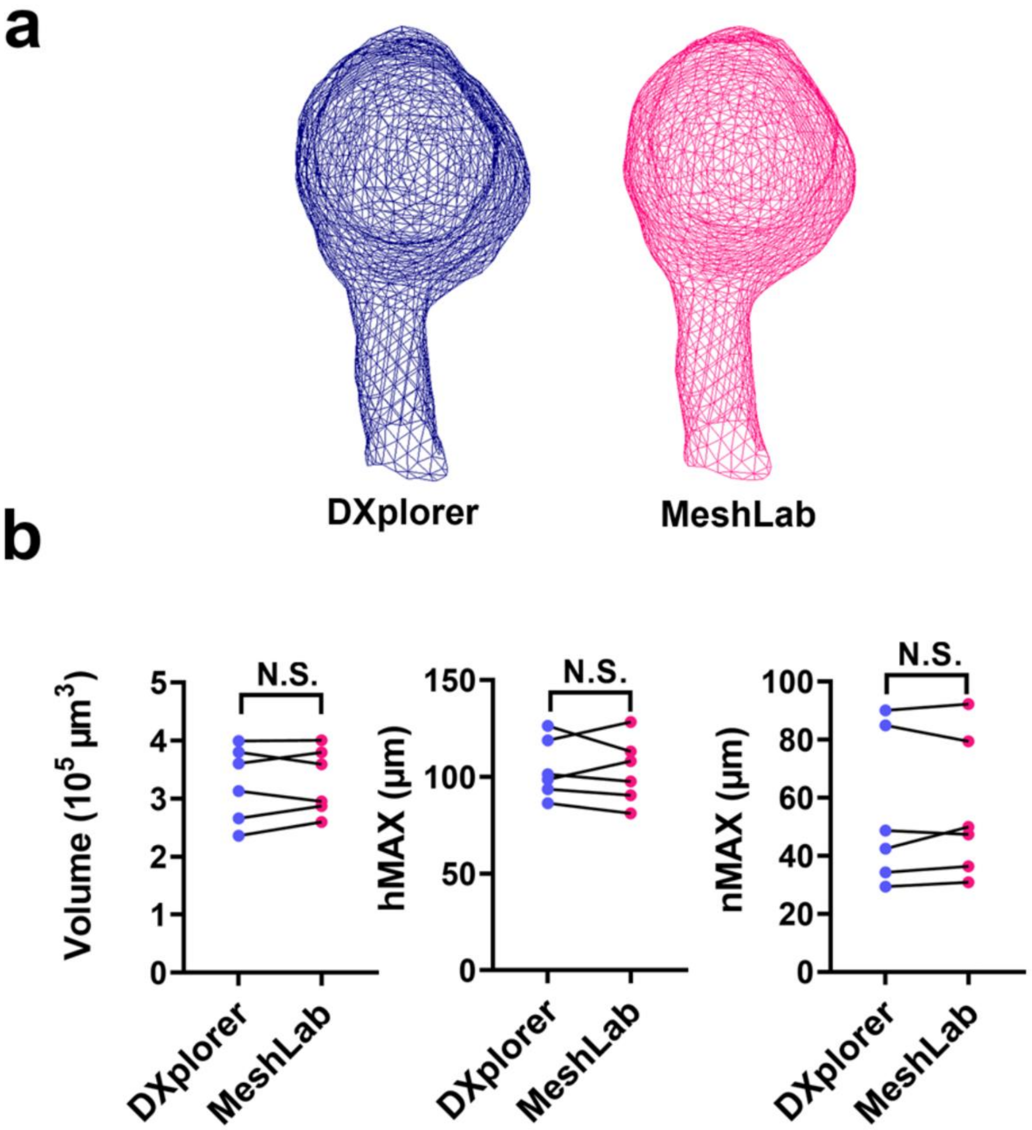

Supplementary Figure 8. Comparison of 3D features analyzed by DXplorer (automatic) and MeshLab (manual)

(a) Representative 3D mesh images of individual glomeruli obtained by Dxplorer and MeshLab. (b) Comparison of 3D volume, hMax and nMax values between DXplorer-based automatic and MeshLab-mediated manual analysis (n = 6).

Supplementary Table 1. Refractive index (RI) measurement of OptiMuS.

	1 trial	2 trial	3 trial
**OptiMuS 1**	**1.474**	**1.474**	**1.473**
**OptiMuS 2**	**1.473**	**1.473**	**1.473**
**OptiMuS 3**	**1.472**	**1.471**	**1.472**

Supplementary Table 2. Properties of clearing reagent in current paper.

	OptiMuS	CLARITY	CUBIC	Sca*l*eS	SeeDB2G	FOCM	MACS
**Procedure**	**Simple**	**Complex**	**Complex**	**Complex**	**Complex**	**Simple**	**Simple**
**Fluorescent signal preservation**	**Good**	**Poor**	**Good**	**Moderate**	**Moderate**	**Poor**	**Good**
**Tissue size change**	**No change**	**Increase**	**Increase**	**Increase**	**Mild decrease**	**Decrease**	**Decrease**
**Clearing time (1-mm rat slice)**	**1.5 hr**	**24 hr**	**11 hr**	**18 hr**	**24 hr**	**40 min**	**1 hr**
**Transparency**	++++	++++	++++	++	+	+	+++
**Lipophilic dye compatibility**	**Yes**	**No**	**No**	**No**	**Yes**	**Unknown**	**Yes**
**Reagent color**	**Colorless**	**Colorless**	**Colorless but turn yellow**	**Colorless**	**Colorless**	**Colorless**	**Colorless but turn yellow**
**Reference**	**-**	**1**	**2**	**3**	**4**	**5**	**6**

Supplementary References

1. Chung, K. *et al.* Structural and molecular interrogation of intact biological systems. *Nature* **497**, 332-337 (2013).
2. Susaki, E.A. *et al.* Whole-brain imaging with single-cell resolution using chemical cocktails and computational analysis. *Cell* **157**, 726-739 (2014).
3. Hama, H. *et al.* ScaleS: an optical clearing palette for biological imaging. *Nat Neurosci* **18**, 1518-1529 (2015).
4. Ke, M.T. *et al.* Super-Resolution Mapping of Neuronal Circuitry With an Index-Optimized Clearing Agent. *Cell Rep* **14**, 2718-2732 (2016).
5. Zhu, X. *et al.* Ultrafast optical clearing method for three-dimensional imaging with cellular resolution. *Proc Natl Acad Sci U S A* **116**, 11480-11489 (2019).
6. Zhu, J. *et al.* MACS: Rapid Aqueous Clearing System for 3D Mapping of Intact Organs. *Adv Sci (Weinh)* **7**, 1903185 (2020).
