# Supplementary material for: Optimized single-step optical clearing solution for 3D volume imaging of biological structures
Source: Commun Biol. 2022 May 9;5:431. doi: 10.1038/s42003-022-03388-8 (PMC9085829; doi:10.1038/s42003-022-03388-8)
Supplement: Supplementary file 3 — Description of Additional Supplementary Files [file 42003_2022_3388_MOESM3_ESM.pdf]

Description of Additional Supplementary Files

File name: Supplementary Data 1
Description: Raw data sets used in figures.
