# Supplementary material for: Optimized single-step optical clearing solution for 3D volume imaging of biological structures
Source: Commun Biol. 2022 May 9;5:431. doi: 10.1038/s42003-022-03388-8 (PMC9085829; doi:10.1038/s42003-022-03388-8)
Supplement: Supplementary file 5 — Reporting Summary [file 42003_2022_3388_MOESM5_ESM.pdf]

nature research

Corresponding author(s): Sunghoe Chang

Last updated by author(s): Mar 9, 2022

Reporting Summary

Nature Research wishes to improve the reproducibility of the work that we publish. This form provides structure for consistency and transparency in reporting. For further information on Nature Research policies, see our Editorial Policies and the Editorial Policy Checklist.

Statistics

For all statistical analyses, confirm that the following items are present in the figure legend, table legend, main text, or Methods section.

n/a	Confirmed	
☐	☒	The exact sample size (*n*) for each experimental group/condition, given as a discrete number and unit of measurement
☐	☒	A statement on whether measurements were taken from distinct samples or whether the same sample was measured repeatedly
☐	☒	The statistical test(s) used AND whether they are one- or two-sided<br>*Only common tests should be described solely by name; describe more complex techniques in the Methods section.*
☒	☐	A description of all covariates tested
☐	☒	A description of any assumptions or corrections, such as tests of normality and adjustment for multiple comparisons
☐	☒	A full description of the statistical parameters including central tendency (e.g. means) or other basic estimates (e.g. regression coefficient) AND variation (e.g. standard deviation) or associated estimates of uncertainty (e.g. confidence intervals)
☐	☒	For null hypothesis testing, the test statistic (e.g. *F*, *t*, *r*) with confidence intervals, effect sizes, degrees of freedom and *P* value noted<br>*Give P values as exact values whenever suitable.*
☒	☐	For Bayesian analysis, information on the choice of priors and Markov chain Monte Carlo settings
☒	☐	For hierarchical and complex designs, identification of the appropriate level for tests and full reporting of outcomes
☒	☐	Estimates of effect sizes (e.g. Cohen's *d*, Pearson's *r*), indicating how they were calculated

Our web collection on statistics for biologists contains articles on many of the points above.

Software and code

Policy information about availability of computer code

Data collection	NIS-Elements AR v.5.01.00 (Nikon), ImSpector Pro (Lavision), and image J 1.53c.<br>Regarding DXplorer (Fig 5 e-f), the earlier version of this software was was published at the 2019 IEEE Visualization Conference (VIS) as a short paper [Choi et al., "Interactive Dendritic Spine Analysis Based on 3D Morphological Features." 2019 IEEE Visualization Conference (VIS), p171-175. IEEE, 2019.]. The new version of this software has recently published in the journal IEEE Transactions on Visualization and Computer Graphics (TVCG) and the code was uploaded at (https://github.com/hvcl/SpineAnalysis_public). The stand-alone software will be available from authors on request.
Data analysis	image J 1.53c, GraphPad Prism 8.0. and Imaris 6.0 for 3D rendering of LSFM images

For manuscripts utilizing custom algorithms or software that are central to the research but not yet described in published literature, software must be made available to editors and reviewers. We strongly encourage code deposition in a community repository (e.g. GitHub). See the Nature Research guidelines for submitting code & software for further information.

Data

Policy information about availability of data

All manuscripts must include a data availability statement. This statement should provide the following information, where applicable:

- Accession codes, unique identifiers, or web links for publicly available datasets
- A list of figures that have associated raw data
- A description of any restrictions on data availability

The datasets generated during and/or analyzed during this study are available from the corresponding author upon reasonable request.

Field-specific reporting

Please select the one below that is the best fit for your research. If you are not sure, read the appropriate sections before making your selection.

☒ Life sciences ☐ Behavioural & social sciences ☐ Ecological, evolutionary & environmental sciences

For a reference copy of the document with all sections, see nature.com/documents/nr-reporting-summary-flat.pdf

Life sciences study design

All studies must disclose on these points even when the disclosure is negative.

Sample size	No statistical methods were used to predetermine sample size. Sample size per group was determined from previous publications with similar methodologies.
Data exclusions	No data excluded.
Replication	The experiments were repeated at least three times independently and the number of iterations was indicated in the manuscript. All assays showed similar results.
Randomization	Intentional randomization is not relevant for most of this study. Instead, mice and rats used for the experiments were randomly chosen from colonies. Brain and tissue sections were chosen randomly for each experimental group so that randomization of tissue sections with respect to treatment is inherent to our experimental design.
Blinding	The data in most of the figures were analyzed on a single-blind basis and samples were processed either simultaneously or in parallel. For Fig. 3 and 4, and Supplementary Fig. 5, 6, and 7 data were obtained from the results of a single experimental condition or from the change between before and after treatment in a single experimental group.

Reporting for specific materials, systems and methods

We require information from authors about some types of materials, experimental systems and methods used in many studies. Here, indicate whether each material, system or method listed is relevant to your study. If you are not sure if a list item applies to your research, read the appropriate section before selecting a response.

Materials & experimental systems

n/a	Involved in the study	
☐	☒	Antibodies
☒	☐	Eukaryotic cell lines
☒	☐	Palaeontology and archaeology
☐	☒	Animals and other organisms
☒	☐	Human research participants
☒	☐	Clinical data
☒	☐	Dual use research of concern

Methods

n/a	Involved in the study	
☒	☐	ChIP-seq
☒	☐	Flow cytometry
☒	☐	MRI-based neuroimaging

Antibodies

Antibodies used	Anti-GFAP(Abcam,ab53554) Lot# GR3221771-3<br>Anti-beta III Tubulin (Abcam, ab18207) Lot# GR3259047-2<br>Alexa Fluor 488 Donkey anti-Goat IgG (H+L)(Invitrogen, A11055) Lot# 2211210<br>Alexa Flour 488, F(ab') 2-Goat anti-Rabbit IgG (H+L)(Invitrogen, A11070) Lot# 2251171<br>DyLight 594 labeled Lycopersicon Esculentum (Tomato) Lectin(Vector Labs, DL-1177) Lot# ZF-8026
Validation	All antibodies were purchased from commercial manufacturer. The validation statement can be found on the manufacturer's website. Several dilutions were tested for each antibody.<br>Anti-GFAP (https://www.abcam.com/gfap-antibody-ab53554.html)<br>Anti-beta III Tubulin (https://www.abcam.com/beta-iii-tubulin-antibody-neuronal-marker-ab18207.html)<br>Alexa Fluor 488 Donkey anti-Goat IgG (H+L) (https://www.thermofisher.com/antibody/product/Donkey-anti-Goat-IgG-H-L-Cross-Adsorbed-Secondary-Antibody-Polyclonal/A-11055)<br>Alexa Flour 488, F(ab') 2-Goat anti-Rabbit IgG (H+L) (https://www.thermofisher.com/antibody/product/Goat-anti-Rabbit-IgG-H-L-Cross-Adsorbed-Secondary-Antibody-Polyclonal/A-11070)<br>DyLight 594 labeled Lycopersicon Esculentum (Tomato) Lectin (https://vectorlabs.com/dylight-594-labeled-lycopersicon-esculentum-tomato-lectin-lel-tl.html)

Animals and other organisms

Policy information about studies involving animals; ARRIVE guidelines recommended for reporting animal research

Laboratory animals	6-8-week-old male C57BL/6N mice<br>6-month-old male Thy1-eYFP H-line transgenic mouse<br>8-12-week-old male ChAT-Cre-tdTomato transgenic mouse<br>Embryonic day 13 ,18 Sprague-Dawley fetal rats were used for whole embryo clearing
Wild animals	This study did not involve wild animals.
Field-collected samples	This study did not include field-collected samples.
Ethics oversight	the Institute of Animal Care and Use Committee (IACUC) guidelines of Seoul Nation University (SNU-200904-2-4) and IACUC of the KBRI (IACUC-18-00018)

Note that full information on the approval of the study protocol must also be provided in the manuscript.
